# The Effects of Resistance Exercise Training on Skeletal Muscle Metabolism and Insulin Resistance Development in Female Rodents with Type 1 Diabetes

**DOI:** 10.1155/2024/5549762

**Published:** 2024-02-22

**Authors:** Mitchell J. Sammut, David P. McBey, Amit P. Sayal, C. W. James Melling

**Affiliations:** ^1^School of Kinesiology, Faculty of Health Sciences, Western University, London, ON, Canada; ^2^Department of Physiology & Pharmacology, Schulich School of Medicine & Dentistry, Western University, London, ON, Canada

## Abstract

The etiology of insulin resistance (IR) development in type 1 diabetes mellitus (T1DM) remains unclear; however, impaired skeletal muscle metabolism may play a role. While IR development has been established in male T1DM rodents, female rodents have yet to be examined in this context. Resistance exercise training (RT) has been shown to improve IR and is associated with a lower risk of hypoglycemia onset in T1DM compared to aerobic exercise. The purpose of this study was to investigate the effects of RT on IR development in female T1DM rodents. Forty Sprague Dawley eight-week-old female rats were divided into four groups: control sedentary (CS; *n* = 10), control trained (CT; *n* = 10), T1DM sedentary (DS; *n* = 10), and T1DM trained (DT; *n* = 10). Multiple low-dose streptozotocin injections were used to induce T1DM. Blood glucose levels were maintained in the 4-9 mmol/l range with intensive insulin therapy. CT and DT underwent weighted ladder climbing 5 days/week for six weeks. Intravenous glucose tolerance tests (IVGTT) were conducted on all animals following the six-week period. Results demonstrate that DS animals exhibited significantly increased weekly blood glucose measures compared to all groups including DT (*p* < 0.0001), despite similar insulin dosage levels. This was concomitant with a significant increase in insulin-adjusted area under the curve following IVGTT in DS (*p* < 0.05), indicative of a reduction in insulin sensitivity. Both DT and DS exhibited greater serum insulin concentrations compared to CT and CS (*p* < 0.05). DS animals also exhibited significantly greater glycogen content in white gastrocnemius muscle compared to CS and DT (*p* < 0.05), whereas DT and DS animals exhibited greater p-Akt: Akt ratio in the white vastus lateralis muscle and citrate synthase activity in the red vastus lateralis muscle compared to CS and CT (*p* < 0.05). These results indicate that female rodents with T1DM develop poor glycemic control and IR which can be attenuated with RT, possibly related to differences in intramyocellular glycogen content.

## 1. Background

Type 1 diabetes mellitus (T1DM) is a chronic metabolic disease characterized by the loss of endogenous insulin production resulting from autoimmune-mediated pancreatic beta cell destruction. The significant reduction in systemic circulation of insulin results in hyperglycemia which can lead to the accumulation of advanced glycation end products within bodily tissues and the development of complications such as nephropathy, neuropathy, retinopathy, and cardiovascular disease (CVD) (1). The current standard of care for patients with T1DM consists of exogenous insulin administration to normalize glycemia; specifically, intensive insulin therapy (IIT) which involves the maintenance of blood glucose within the 4-9 mmol/l range (2). While IIT helps individuals with T1DM to avoid prolonged hyperglycemia, insulin resistance (IR) is a prominent feature in approximately 20-30% of patients treated with IIT (3–7). Termed “double diabetes,” the presence of IR in T1DM significantly increases the risk of diabetes-related complications such as CVD (5, 8–13).

While the etiology of IR in T1DM is currently unknown, it has been shown to be distinct from that of other dysfunctional metabolic states such as metabolic syndrome and type 2 diabetes mellitus (T2DM) (14). Indeed, IR is present in otherwise healthy and normal-weight adolescents and young adults with T1DM and is not explained by common predictive factors such as body mass index, plasma lipids, reduced physical activity levels, and visceral adiposity (15–18).

Skeletal muscle is responsible for ~80% of postprandial glucose uptake, making this tissue an important determinant of whole-body insulin sensitivity (19). Furthermore, skeletal muscle is a heterogeneous tissue consisting of oxidative slow-twitch/type I (red) and glycolytic fast-twitch/type IIA/type IIB (white) myofibers. Importantly, red and white muscle tissues exhibit different metabolic characteristics which influence insulin-action (e.g., differences in capillary density and mitochondrial content), such that slow-twitch fibers typically exhibit greater insulin sensitivity (20). Unlike in otherwise healthy individuals, the peripheral administration of insulin bypasses the natural flow of insulin through the liver. That is, skeletal muscle becomes the “first-pass” tissue for insulin and is the primary tissue for managing dysglycemia in T1DM (21–23). Previous research has reported impairments in skeletal muscle metabolism in humans with T1DM including mitochondrial dysfunction and muscle fiber type alterations (24–32). Specifically, mitochondrial impairments in skeletal muscle have been shown to relate to IR in patients with T1DM (29, 30). Considering the important “first-pass” role of skeletal muscle in T1DM, impairments in substrate oxidation in this tissue likely lead to the impairment of glycemic control and insulin sensitivity in T1DM (22). Importantly, it has been shown that ~30-40% of glucose is oxidized by skeletal muscle following an oral glucose tolerance test (33) and that skeletal muscle oxidative capacity is a superior predictor of insulin sensitivity compared to intramyocellular lipid status (34). Our laboratory has shown that sedentary male T1DM rodents with IR exhibit reduced skeletal muscle oxidative capacity (35). Therefore, skeletal muscle oxidation of glucose may be an important determinant of insulin sensitivity in T1DM; however, the exact cellular mechanisms governing IR in T1DM remain unclear.

Exercise training has been shown to significantly increase insulin sensitivity and improve glucose homeostasis in patients with T1DM and animal models of this disease (35–43). Unfortunately, individuals with T1DM are largely inactive due to the fear of hypoglycemia, which is one of the largest barriers to exercise in this population, especially for those with IR (44–46). Aerobic exercise is associated with hypoglycemia onset in T1DM, characterized by a significant drop in blood glucose below 3.9 mmol/l (45, 47–49). Conversely, resistance exercise has been shown to be associated with a lower risk of exercise-induced hypoglycemia in patients with T1DM (45, 50, 51). Therefore, resistance exercise may be a safe and effective exercise intervention to improve IR in patients with T1DM.

Most work investigating IR in T1DM from a mechanistic standpoint has been conducted solely in males (35, 38–40, 52), while very limited work has been conducted in females (53, 54). The investigation of females with T1DM is warranted as it has been shown that sex differences exist in the metabolic effects of exercise (55, 56). Indeed, it has been shown that female adults with T1DM exhibit differential alterations of mitochondria including lower oxidative capacity and greater skeletal muscle IR compared to their male counterparts (25, 27, 57, 58). Moreover, no study has assessed the effects of resistance exercise training (RT) on IR development in female rodents with T1DM.

The purpose of this study was to examine the effects of six weeks of RT on IR development in female rodents with T1DM. It was hypothesized that IR in female rodents with T1DM would be concomitant with impairments in skeletal muscle mitochondrial capacity and that six weeks of RT would ameliorate IR and improve the capacity of mitochondria in skeletal muscle.

## 2. Materials and Methods

### 2.1. Ethics Approval

The protocols utilized in this study were approved by the University Council of Animal Care of Western University (London, Ontario, Canada) in accordance with the standards of the Canadian Council on Animal Care.

### 2.2. Animals

Forty female Sprague Dawley rats were obtained from Charles River Laboratories (St. Constant, Que., Canada) at eight weeks of age. All rats were caged in pairs and housed in a room with a 12-hour light-dark cycle, a temperature of 20.5°C, and relative humidity of 40%. Rats were provided access to standard rat chow and water ad libitum over the course of the study.

### 2.3. Experimental Groups

Rats were randomly assigned into one of four groups: control (non-T1DM) sedentary (CS; *n* = 10), control resistance trained (CT; *n* = 10), T1DM sedentary (DS; *n* = 10), and T1DM resistance trained (DT; *n* = 10).

### 2.4. Experimental Procedures

#### 2.4.1. T1DM Induction and Insulin Pellet Implantation

Upon arrival, all rats underwent an acclimatization process for one week. Following this, the two T1DM groups (DS and DT) underwent seven consecutive days of intraperitoneal low-dose injections of streptozotocin (STZ; Sigma-Aldrich) to induce T1DM. Over the seven days, 20 mg/kg of STZ dissolved in citrate buffer (0.1 M, pH 4.5) was injected within fifteen minutes of solution preparation. Diabetes was confirmed following the seven days of STZ injections by two nonfasting blood glucose measurements of ~11 mmol/l or greater (59). After confirmation of diabetes induction, one insulin pellet (2 IU insulin/day) was surgically implanted subcutaneously into the abdominal region. The insulin dose was modified over the course of the study via the removal of a portion of the insulin pellet if required for appropriate blood glucose maintenance. Blood glucose was intended to be maintained between 4 and 9 mmol/l for each T1DM rat throughout the study.

#### 2.4.2. Exercise Training

RT consisted of vertical ladder climbing with a weight-loaded bag secured to the proximal portion of the tail. Prior to week one of RT, CT and DT animals underwent two separate familiarization sessions consisting of 10 consecutive ladder climbs at 5, 15, 20, and 35% of their body weight (2-3 climbs at each weight). This allowed rats to become acquainted with the process of ladder climbing before the commencement of training. Rats were allowed to rest in a dark box at the top of the ladder for ~1-2 minutes in-between climbs. An initial maximal carrying capacity was determined during the first day of week one of training for each rat. This was determined by beginning each rat at a carrying load equal to 75% of their body weight and progressively adding 10-30 g until failure was reached (defined as an unwillingness to climb despite hind limb stimulation via touch and air bursts). Following the determination of maximal carrying capacity, training periods consisted of rats performing four consecutive climbs at 50, 75, 90, and 100% of their maximal carrying capacity, followed by as many climbs as possible until failure at 100% maximal carrying capacity. A new maximal carrying capacity was established every four days by starting with each rat's previous maximal capacity and gradually adding 10-30 g until failure, following the progressive overload principle. CT and DT rats performed RT five days/week for six weeks.

### 2.5. Experimental Measures

#### 2.5.1. Body Weights and Blood Glucose

The body weight of each rat was measured and recorded once per week over the course of the study. Weekly nonfasting blood glucose was also measured from a small blood droplet (~50 *μ*l) obtained from the saphenous vein using the Freestyle Lite Blood Glucose Monitoring System (Abbott Diabetes Care, INC.).

#### 2.5.2. Intravenous Glucose Tolerance Test

Intravenous glucose tolerance tests (IVGTTs) were performed for all rats following the 6-week training period. Following a 4-12 hour fast, a baseline blood glucose measurement was obtained. Shorter fasting periods were used for DS and DT (~4 hours) compared to non-T1DM control (~12 hours) animals to avoid hypoglycemic episodes. A sterile dextrose solution (10%; 5 g dextrose in 50 ml ddH_2_O) was injected (2 ml/kg) into the tail vein of the conscious rats. Blood glucose was then measured in 10-minute intervals up to 40 minutes postinjection. The area under the curve (AUC) for the IVGTT was determined 40 minutes postdextrose injection.

#### 2.5.3. Blood and Tissue Collection

Rats were sacrificed immediately following the IVGTT during the final week of the study. Sacrifice was performed via anaesthetization with isoflurane, followed by cardiac exsanguination. Approximately three blood samples of 500 *μ*l were collected from each rat. Blood samples were centrifuged for 30 minutes at 3000 rpm, and serum was then transferred to 1.5 ml Eppendorf tubes. The lower limbs were dissected, and the vastus lateralis (red and white), gastrocnemius (red and white), and soleus muscles were removed and immediately frozen in liquid nitrogen. Tissues and serum were then stored at -80°C until later analysis.

#### 2.5.4. Muscle Glycogen Content

Red and white gastrocnemius muscle (~20 mg) was homogenized in 30% KOH saturated with Na_2_SO_4_ and boiled for 30 minutes (47). Glycogen in muscle samples was precipitated with 95% ethanol, and samples were left to rest on ice for an additional 30 minutes. Samples were then centrifuged for 20-30 minutes at 3000 rpm. After centrifugation, the supernatant was discarded, and glycogen pellets were immediately resuspended in 3 ml of ddH_2_O. Glycogen pellets were placed on ice until a homogenous solution resulted and then split into three 1 ml glass tubes for triplicate analysis. One ml of 5% phenol and 5 ml of sulfuric acid (96-98%) were subsequently added to each tube and allowed to stand for 5 minutes at room temperature and then incubated at 25-30°C for 10 minutes. The colour reaction of samples was analyzed using a spectrophotometer at a wavelength of 490 nm.

#### 2.5.5. *β*-Oxidation Activity

Approximately 30 mg of soleus muscle was excised and placed in a 1.5 ml Eppendorf tube. Sample buffer (5 mM K_2_HPO_4_, 1 mM EDTA, 0.1 mM DTT; pH of 7.4) was added to soleus muscle samples for a 1 : 10 weight-volume (w/v) ratio of tissue to buffer. Submerged tissue samples were homogenized with three, 1-3 second pulses by a basic mechanical homogenizer (IKA Laboratories). Homogenized soleus muscle samples were added to an Eppendorf tube with assay buffer (1 M Tris-HCl, pH 7.0, 0.5 M EDTA, pH 8.0, 10% Triton X-100) and 5 mM nicotinamide adenine dinucleotide (NADH). Sample Eppendorf tubes were incubated for 4 minutes at 30°C to permeabilize mitochondria. Following incubation, the sample mixture was transferred to a cuvette, and the reaction was initiated by adding 5 mM acetoacetyl CoA (60). The sample cuvette was vortexed, and the reaction was read for 2 minutes in 30-second intervals on a NanoDrop2000 C Spectrophotometer (Waltham, MA, USA) at 340 nm.

#### 2.5.6. Citrate Synthase Activity

Citrate synthase activity was measured in the red vastus lateralis muscle using an assay kit (#ab239712, Abcam, Cambridge, MA, USA). Approximately 10 mg of red vastus lateralis muscle was excised and placed in a 1.5 ml Eppendorf tube. Citrate synthase assay buffer was added to red vastus samples for a 1 : 10 weight-volume (w/v) ratio of tissue to buffer. Submerged tissue samples were homogenized with three, 1-3 second pulses by a basic mechanical homogenizer (IKA Laboratories, Wilmington, NC, USA). On a 96-well plate, 5 *μ*l of sample was added to wells with reaction buffer, and the rate of absorbance change was read using a microplate reader at 412 nm at 13-second intervals over 6.5 minutes.

#### 2.5.7. Western Blot

Red and white vastus lateralis muscle (20 mg) was submerged 1 : 20 (w/v) in lysis buffer (15 mM Tris pH = 7.0, 600 mM NaCl, and 0.1 mM EDTA). The submerged tissue was homogenized with three, 1-3 second pulses using a basic mechanical homogenizer (IKA Laboratories, Wilmington, NC, USA). Tissue homogenate was transferred into Eppendorf tubes (1.5 ml) and agitated on a shaker for 2 hours on ice. Samples were then centrifuged at 12,000 rpm for 20 minutes at 4°C. Sample supernatant was extracted and transferred to a different Eppendorf tube (1.5 ml) and stored at -80°C. Total sample protein concentration and loading volumes were determined using a Bradford protein assay (61). In Eppendorf tubes, protein samples mixed with an equal volume of 2x Laemmli SDS-PAGE (4% SDS, 20% glycerol, 10% *β*-mercaptoethanol, 0.015% bromophenol blue, 0.125 M Tris, pH 6.8) were subsequently boiled at 90°C in a water bath for 5 minutes. Twenty *μ*g of the sample protein was then loaded into 12% polyacrylamide gels and ran at 75-150 V for 2 hours. Transfer of gel protein to nitrocellulose membranes (Bio-Rad, Mississauga, ON, Canada) was conducted at 70 V for 90 minutes. Following completion of the transfer, membranes were stored at 4°C in TBS-T (Tris Buffer Saline, 0.1% Tween-20), overnight. The following morning, membranes were washed in fresh TBS-T for 5 minutes. Following washing, membranes were blocked with a 5% w/v solution of TBS-T and skim milk powder or bovine serum albumin (BSA) for 1-2 hours. Blocked membranes were then washed for 5 minutes in TBS-T. Washed membranes were incubated for 2 hours at room temperature with primary antibodies detecting: Akt (Cell Signalling; 4691, Danvers, MA, USA), phosphorylated (ser473) Akt (Cell Signalling; 4060, Danvers, MA, USA), and mitochondrial complexes (Abcam; ab110413, Cambridge, MA, USA). Following incubation, the primary antibody was removed, and membranes were subsequently washed in TBS-T for 10 minutes and repeated for three washes. Membranes were then incubated in a 5% w/v solution of TBS-T and skim milk powder or BSA, and secondary antibody (#170-6515 goat anti-rabbit IgG HRP conjugate, #170-6516 goat anti-mouse IgG HRP conjugate; Bio-Rad, 57 Hercules, CA, USA) at a 1 : 20000 dilution for 2 hours at room temperature. After incubation, membranes were washed for 10 minutes in TBS-T and repeated for three washes. Membranes were then treated with Bio-Rad chemiluminescence substrate, and the images were subsequently captured using the Bio-Rad ChemiDoc MP System. Western blot image quantification was conducted using ImageJ.

#### 2.5.8. Insulin and Estradiol Quantification

Insulin and estradiol serum concentrations were determined using enzyme-linked immunosorbent assay (ELISA) kits (ELISA; Rat Insulin ELISA Kit, ALPCO; 17-*β* Estradiol ELISA Kit, Abcam). Blanks, standards, and serum samples were added to a 96-well microplate precoated with anti-insulin or anti-estradiol IgG. Insulin or 17-*β* estradiol-HRP conjugate was added to each well, and plates were incubated for 2 hours at 25-37°C. Following incubation, plates were aspirated and washed three to six times with wash buffer. The substrate solution was then added to each well and further incubated for 15-30 minutes. Following substrate incubation, stop solution was added to each well, and plates were immediately read at 450 nM using a microplate reader. The absorbance values of samples were adjusted based on the absorbance of blanks. Hormone concentration was interpolated from a standard curve generated from the blank and standards.

### 2.6. Data Analysis

GraphPad Prism 8 (GraphPad Software, Inc.) was used to complete statistical data analysis. Weekly blood glucose and body mass measures were analyzed using a three-way analysis of variance (ANOVA) with time, diabetes, and exercise training as factors. Maximal carrying capacity, serum estradiol and insulin concentrations, IVGTT AUC and skeletal muscle content (glycogen, Akt, p-Akt, and mitochondrial complexes), and enzyme activity (*β*-oxidation and citrate synthase) were analyzed using a two-way ANOVA with diabetes and exercise training as factors. Bonferroni's multiple comparisons test was used for post hoc analysis when significant differences were observed. Statistical significance was accepted at an alpha value of 0.05. Correlational analysis of IVGTT AUC and serum estradiol was completed using linear regression.

## 3. Results

### 3.1. Animal Characteristics

**Figure 1:**
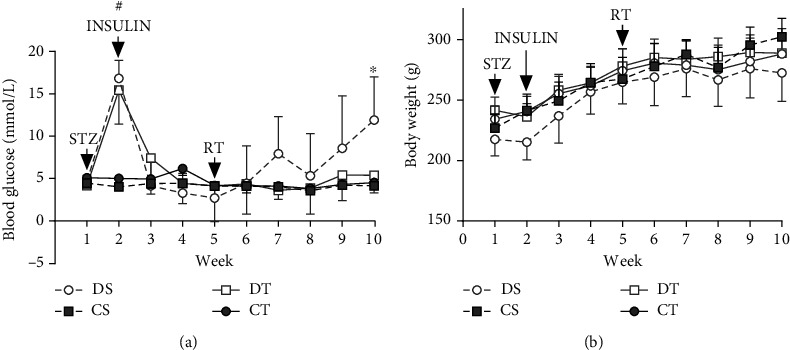
Weekly blood glucose (a) and body weight (b) data. STZ denotes the start of streptozotocin injections to induce T1DM in DS and DT animals. Insulin denotes the start of insulin treatment for DS and DT animals. RT denotes the start of the resistance training regimen for CT and DT animals. (a) Mean weekly nonfasting blood glucose measures (mmol/l). Data is presented as mean ± SD. # denotes a significant difference between T1DM and control animals. ∗ denotes a significant difference between DS and all other groups. (b) Mean weekly body weight measures (g). Data is presented as mean ± SD. No differences were observed between groups within any timepoint. Abbreviations: RT: resistance training; STZ: streptozotocin.

Weekly nonfasting blood glucose (Figure 1(a)) and body mass (Figure 1(b)) measures were analyzed to examine and maintain the health and glycemic control of each animal. One animal from the DS group met its endpoint prematurely and did not contribute to the data. For blood glucose, main effects of diabetes (*F* (1, 343) = 64.11, *p* < 0.0001) and time (*F* (9, 343) = 23.25, *p* < 0.0001) were statistically significant. There was a significant interaction between time and diabetes (*F* (9, 343) = 22.93, *p* < 0.0001), time and training (*F* (9, 343) = 3.799, *p* < 0.05), diabetes and training (*F* (1, 343) = 10.66, *p* < 0.05) and time, and diabetes and training (*F* (9, 343) = 3.062, *p* < 0.05). During week 2 of the study, DS and DT had significantly higher blood glucose levels compared to CT and CS (*p* < 0.0001), while DS and DT blood glucose did not differ (*p* > 0.05). During week 10, DS blood glucose was significantly greater than all groups (*p* < 0.0001). For body weight, main effects of diabetes (*F* (1, 345) = 8.059, *p* < 0.05), time (*F* (9, 345) = 59.86, *p* < 0.0001), and training (*F* (1, 345) = 16.61, *p* < 0.0001) were statistically significant, and there was a significant interaction between diabetes and training (*F* (1, 345) = 26.70, *p* < 0.0001). These results indicate that DS animals exhibited a relative reduction in glycemic control over the course of the study period compared to control and trained T1DM animals.

### 3.2. Carrying Capacity

**Figure 2:**
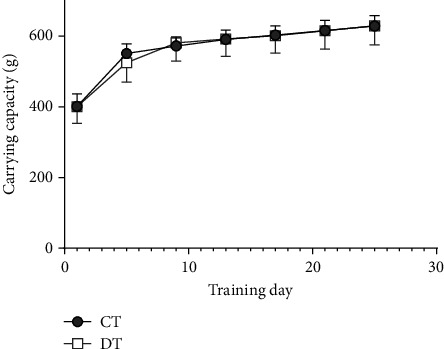
Maximal carrying capacity (g) for animals undergoing RT. Data is presented as mean ± SD. No differences were observed between groups.

Carrying capacities of CT and DT (Figure 2) were recorded and analyzed to ensure that similar loads were carried by each group throughout the training period. A main effect of training day was statistically significant (*F* (6, 126) = 71.21, *p* < 0.0001). No significant differences (*p* > 0.05) were observed in carrying capacity loads (expressed in grams) between CT and DT over the course of the study. Therefore, differences in outcome measures between CT and DT are likely not attributable to different training stimuli.

### 3.3. Serum Insulin and Estradiol Concentration

**Figure 3:**
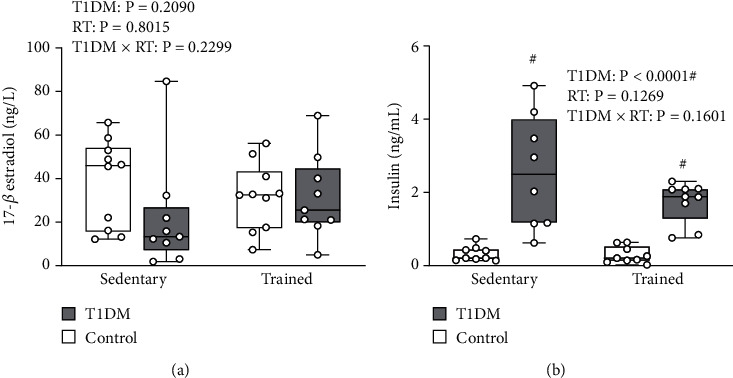
Serum estradiol (a) and serum insulin (b) concentrations. (a) Mean serum estradiol (ng/l). Data is presented as mean ± SD. No differences were observed between groups. (b) Mean serum insulin (ng/ml). Data is presented as mean ± SD. # denotes a significant difference between T1DM and control rodents. Abbreviations: RT: resistance training; T1DM: type 1 diabetes mellitus.

To determine the hormonal influence on glucose metabolism, we next measured the concentrations of circulating 17-*β* estradiol and insulin from serum samples taken immediately after the IVGTT during animal sacrifice. No differences in serum estradiol were observed between groups (*p* > 0.05) (Figure 3(a)). Serum insulin exhibited a main effect of diabetes (*F* (1, 31) = 44.99, *p* < 0.0001) (Figure 3(b)). DS (*p* < 0.0001) and DT (*p* < 0.05) serum insulin values were significantly greater than CS and CT animals, while DS and DT serum insulin values did not differ significantly (*p* > 0.05). Elevated serum insulin concentrations in both T1DM groups compared to control animals suggest a state of hyperinsulinemia in these animals likely as a result of exogenous insulin treatment.

### 3.4. Intravenous Glucose Tolerance Test

**Figure 4:**
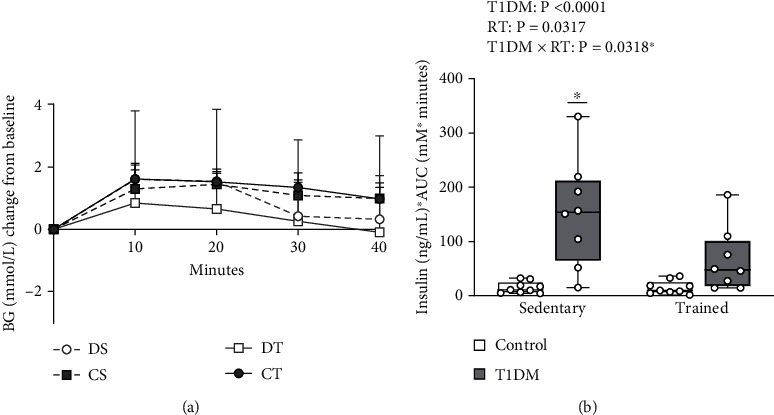
IVGTT blood glucose (a) and insulin-adjusted IVGTT AUC (b). (a) Mean blood glucose measures 40 minutes postdextrose injection (mmol/l). Data is presented as mean ± SD. (b) Mean insulin- (ng/ml) adjusted IVGTT AUC (mM∗minutes). Data is presented as mean ± SD. ∗ denotes a significant difference between DS and all other groups. Abbreviations: AUC: area under the curve; BG: blood glucose; RT: resistance training; T1DM: type 1 diabetes mellitus.

Blood glucose measures during the IVGTT (Figure 4(a)) were used to determine AUC as an indicator of insulin sensitivity. IVGTT AUC (Figure 4(b)) was then adjusted for serum insulin concentration to gain a more accurate measure of glucose clearance. For insulin-adjusted IVGTT AUC, there was a main effect of diabetes (*F* (1, 30) = 23.52, *p* < 0.0001) and training (*F* (1, 30) = 5.078, *p* < 0.05), and a significant interaction between diabetes and training (*F* (1, 30) = 5.072, *p* < 0.05). DS exhibited significantly greater insulin-adjusted IVGTT AUC compared to CS (*p* < 0.0001), CT (*p* < 0.0001), and DT (*p* < 0.05), indicative of IR in these animals. As hypothesized, this suggests that RT prevented IR development in female T1DM rodents.

### 3.5. Correlation between Serum Estradiol Concentration and Insulin-Adjusted IVGTT AUC

**Figure 5:**
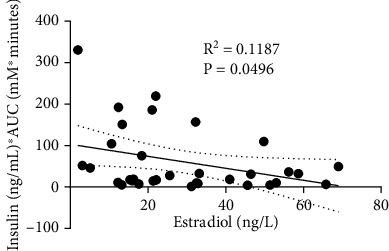
Linear regression between insulin- (ng/ml) adjusted IVGTT AUC (mM∗minutes) and serum estradiol (ng/l). Abbreviations: AUC: area under the curve.

As 17-*β* estradiol may influence insulin sensitivity in females, we conducted a correlational analysis between serum estradiol concentration and insulin-adjusted IVGTT AUC. While significantly nonzero (*F* (1, 31) = 4.176, *p* < 0.05), linear regression analysis revealed a limited explanation of the variance in insulin sensitivity measures by serum estradiol (*R*^2^ = 0.1187) when values from all groups were pooled (Figure 5). This result persisted when data was segregated by group (Supplemental Figure 1). This suggests that the influence of estradiol concentration on glucose clearance in these animals was negligible.

### 3.6. Muscle Glycogen Content, *β*-Oxidation Activity, and Citrate Synthase Activity

**Figure 6:**
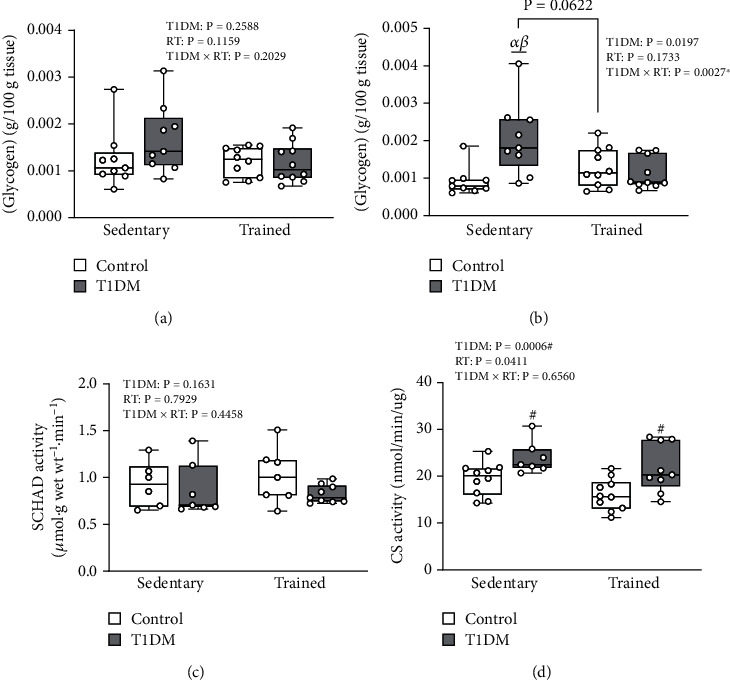
Red gastrocnemius muscle glycogen content (a), white gastrocnemius muscle glycogen content (b), soleus muscle SCHAD activity (c), and red vastus lateralis muscle citrate synthase activity (d). (a) Mean red gastrocnemius muscle glycogen content (g/100 g tissue). Data is presented as mean ± SD. No differences were observed between groups. (b) Mean white gastrocnemius muscle glycogen content (g/100 g tissue). Data is presented as mean ± SD. *α* denotes a significant difference between DS and CS. *β* denotes a significant difference between DS and DT. A difference between DS and CT approached significance (*p* = 0.0622). (c) Mean soleus muscle SCHAD activity (*μ*mol·g wet wt-1·min-1). Data is presented as mean ± SD. No differences were observed between groups. (d) Mean red vastus lateralis muscle citrate synthase activity (nmol/min/*μ*g). Data is presented as mean ± SD. # denotes a significant difference between T1DM and control animals. Abbreviations: CS: citrate synthase; RT: resistance training; SCHAD: short-chain *β*-hydroxyacyl-CoA dehydrogenase; T1DM: type 1 diabetes mellitus; wt: weight.

We next aimed to investigate the metabolism of skeletal muscle following a glucose challenge using muscle samples obtained immediately post-IVGTT. A series of assays were conducted to measure intramyocellular glycogen content, lipid metabolism, and oxidative capacity as these represent important determinants of insulin sensitivity and glucose uptake. Red gastrocnemius muscle glycogen content (Figure 6(a)) did not differ between groups (*p* > 0.05), whereas glycogen content in the white gastrocnemius muscle (Figure 6(b)) exhibited a main effect of diabetes (*F* (1, 34) = 5.989, *p* < 0.05) and a significant interaction between diabetes and training (*F* (1, 34) = 10.49, *p* < 0.05). DS animals exhibited significantly greater glycogen content in white gastrocnemius muscle compared to CS and DT (*p* < 0.05), whereas DS animals approached significance compared to CT (*p* = 0.0622). Soleus muscle *β*-oxidation and short-chain *β*-hydroxyacyl-CoA dehydrogenase (SCHAD) activity (Figure 6(c)) did not differ between groups (*p* > 0.05). Red vastus lateralis muscle citrate synthase activity (Figure 6(d)) exhibited a main effect of diabetes (*F* (1, 32) = 14.59, *p* < 0.05) and training (*F* (1, 32) = 4.532, *p* < 0.05). Contrary to our hypothesis, the results of these assays suggest that insulin-insensitive DS animals do not exhibit impairments in skeletal muscle oxidative capacity, rather alterations in intramyocellular glycogen content which was prevented with RT.

### 3.7. Muscle Insulin Signalling Activation and Mitochondrial Enzyme Content

**Figure 7:**
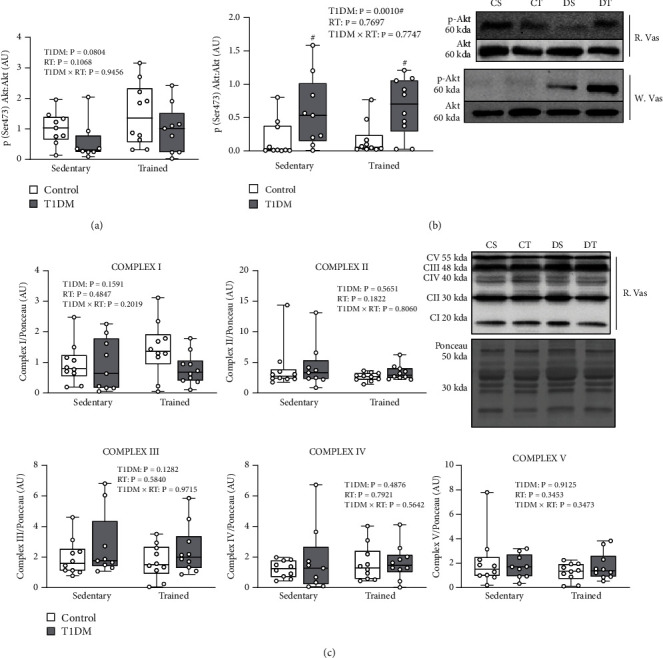
Red vastus lateralis muscle p-Akt: Akt ratio (a), white vastus lateralis muscle p-Akt: Akt ratio (b), red vastus lateralis muscle OXPHOS protein content (c). (a) Mean red vastus lateralis muscle p-Akt: Akt ratio. Data is presented as mean ± SD. No differences were observed between groups. (b) Mean white vastus lateralis muscle p-Akt: Akt. Data is presented as mean ± SD. # Denotes a significant difference between T1DM and control animals. (c) Mean red vastus lateralis muscle OXPHOS protein content (AU/Ponceau). Data is presented as mean ± SD. No differences were observed between groups. Abbreviations: AU: arbitrary units; RT: resistance training; R. Vas: red vastus lateralis muscle; T1DM: type 1 diabetes mellitus; W. Vas: white vastus lateralis muscle.

Akt (protein kinase B) activation via phosphorylation was assessed to determine insulin sensitivity at the level of distal intracellular insulin signalling in skeletal muscle. In the red vastus lateralis muscle, the ratio of p-Akt(ser473) to total Akt (Figure 7(a)) approached significance for a main effect of diabetes (*p* = 0.0804) but did not significantly differ between groups (*p* > 0.05). In the white vastus lateralis muscle, the ratio of p-Akt(ser473) to total Akt (Figure 7(b)) exhibited a main effect of diabetes (*F* (1, 35) = 13.02, *p* < 0.05). Red vastus lateralis muscle oxidative phosphorylation (OXPHOS) protein content of complexes I-V (Figure 7(c)) did not significantly differ between groups (*p* > 0.05). Protein content was normalized to Ponceau staining of membranes. This suggests that Akt activation and mitochondrial content in skeletal muscle were not impaired in DS animals exhibiting IR and were not altered with RT.

## 4. Discussion

The primary finding of the current study is that, similar to their male counterparts (35, 38–40, 52), female rodents with T1DM develop IR. By the end of the study, DS animals exhibited nonfasting blood glucose measures significantly greater than all groups. Additionally, IVGTT AUC was significantly greater in DS animals, indicative of a reduction in insulin sensitivity. To our knowledge, this would be the first evidence that insulin-treated female rodents with well-controlled T1DM develop IR. DT animals exhibited reduced blood glucose levels and lower IVGTT AUC compared to DS, which indicates that six weeks of RT in female rodents with T1DM were able to prevent IR development. These findings are in line with our previous report in male T1DM rodents (38), and literature reporting RT-induced improvements in insulin sensitivity in individuals with T2DM, and in both males and females (62–68).

Our analyses of skeletal muscle tissue revealed distinct alterations in intramyocellular glycogen storage in sedentary and trained rodents with T1DM. Specifically, DS animals exhibited an elevation in white gastrocnemius muscle glycogen content. This may have been a result of the elevated levels of serum insulin observed in T1DM animals in this study, as insulin plays an important role in promoting intramuscular glycogen synthesis (69). Indeed, patients with T1DM exhibit insulin levels ~2.5 times greater than non-T1DM individuals with similar glycemia (21). Subcutaneous insulin delivery bypasses the liver (and the first-pass hepatic insulin extraction) and results in elevated levels of systemic insulin in T1DM. Termed “peripheral hyperinsulinemia,” elevated levels of systemic insulin have been implicated in the development of IR in this population (21, 70). Regardless of training status, T1DM rodents exhibited elevated Akt activation in the white vastus lateralis muscle during the IVGTT. Insulin-stimulated Akt activation has been shown to play an important role in glucose uptake and incorporation into glycogen in muscle cells (71). Despite elevated serum insulin and Akt activation in both T1DM groups, only DS animals exhibited increased intramyocellular glycogen content. These findings may provide mechanistic insights into IR development in T1DM: (1) IR in skeletal muscle in T1DM may develop downstream of Akt activation and (2) elevated intramyocellular glycogen content may play a negative role on insulin sensitivity. In support of the latter, it has been shown that high muscle glycogen content significantly reduces insulin-stimulated glucose uptake in white gastrocnemius muscle (72). However, this reduction in insulin sensitivity in white muscle with high glycogen content was accompanied by reduced insulin-stimulated Akt activation (72). The assimilation into glycogen is a major pathway for insulin-stimulated glucose disposal in skeletal muscle and is tightly regulated to avoid glycogen overaccumulation. As such, glycogen content is inversely related to glycogen synthase (GS) activity such that elevated glycogen levels further inhibit glycogen synthesis via a reduction in GS activity (73–77). In humans without diabetes, chronic hyperglycemia via glucose infusion has been shown to increase intramyocellular glycogen content, reduce insulin-stimulated GS activity, and reduce insulin sensitivity (78). The mechanisms leading to elevated glycogen content in white gastrocnemius muscle in DS animals are unclear; however, it is plausible that hyperinsulinemia led to an increase in fast-twitch muscle glucose uptake, which resulted in elevated glycogen content. It has been shown that glycolytic fast-twitch muscle fibers exhibit a greater capacity to store glycogen compared to oxidative slow-twitch muscle fibers (79), which in turn may explain why elevated glycogen content was not observed in red gastrocnemius muscle. In support of this theory, we have previously reported elevated glycogen content in type IIa fibers in male T1DM rodents treated with IIT (52). While few studies have directly compared muscular glycogen content in humans with and without T1DM, they did not observe differences between patients with T1DM treated with IIT and non-T1DM individuals (80, 81). However, these studies used noninvasive magnetic resonance spectroscopy and did not distinguish between glycogen in red versus white muscle tissue. This suggests that alterations in muscle glycogen storage with T1DM may be fiber-specific, as has been previously reported in patients with T2DM (82).

In the current study, the increase in intramyocellular glycogen content observed in DS animals was not found in DT animals, suggesting that RT prevented this metabolic abnormality. Following training, it is plausible that more glucose entering the skeletal muscle of DT animals would be diverted towards anabolic and biosynthetic pathways to support RT-induced muscle hypertrophy. This in turn would reduce glycogenesis flux and prevent elevated muscle glycogen content. Moreover, it has been shown that fast-twitch muscle fibers preferentially develop hypertrophy with high-intensity RT in females (83, 84), which would support our observations of reduced fast-twitch but not slow-twitch muscle glycogen content in DT compared to DS animals. Based on observational and isotope tracer analysis, it has recently been theorised that intracellular glucose and glycolytic intermediates are incorporated into muscle proteins and may support the process of hypertrophy via the pentose phosphate, serine synthesis, and hexosamine pathways (85). Furthermore, additional work is warranted to better understand the mechanisms governing RT-induced improvements in IR and the relationship between muscle glycogen content and anabolic pathways in T1DM.

In the current investigation, citrate synthase activity was elevated in both DS and DT animals despite no differences in mitochondrial enzyme protein content compared to non-T1DM control rodents. This suggests that T1DM rodents exhibited alterations in intrinsic mitochondrial capacity rather than mitochondrial content, which is supported by data in young adults with T1DM (24). This finding contrasts our hypothesis and previous findings that citrate synthase activity is reduced in male T1DM rodents (35) as well as other reports of impaired skeletal muscle mitochondrial function in male and female patients with T1DM (24, 25, 27–30, 58). In alignment with our observations, studies in human patients with T1DM and STZ-induced T1DM rodents have reported enhanced intrinsic mitochondrial OXPHOS capacity (27, 86). An explanation for our observation may be that T1DM rodents exhibited a compensatory increase in muscle oxidative capacity to manage the increased intracellular glucose load during the IVGTT. Indeed, experimental chronic hyperinsulinemia in healthy humans has been shown to significantly reduce insulin-stimulated glucose utilization while enhancing oxidative glucose disposal in skeletal muscle (87). It has previously been shown that young women with T1DM do not exhibit impairments in in vivo muscle mitochondrial oxidative capacity compared to age-matched women without T1DM (88). In combination with our findings, these data may suggest that impairments in mitochondrial oxidative capacity in skeletal muscle are not implicated in the development of IR in females with T1DM. However, the mechanistic relationship between skeletal muscle mitochondrial capacity and IR development in female patients with T1DM remains unclear and requires further investigation.

Additionally, we observed a reduction in citrate synthase activity with RT in T1DM and non-T1DM control rodents, which may appear counterintuitive to our previous findings of improved skeletal muscle oxidative capacity following aerobic and combined (aerobic and resistance) exercise training in male T1DM rodents (35). However, our findings are in line with multiple investigations reporting reductions in citrate synthase activity with RT (89). A potential explanation for this phenomenon is a “dilution” of the mitochondrial pool with RT-induced muscle fiber hypertrophy, i.e., mitochondrial biogenesis with RT occurs at a slower rate compared to increases in muscle cell volume, resulting in a reduction in citrate synthase activity per unit of muscle (89). Further work is needed to understand the morphological changes in skeletal muscle in T1DM as a result of RT and its impact on the oxidative capacity of the muscle.

Our laboratory has previously shown increases in insulin-desensitizing lipid intermediates, such as diacylglycerol (90), in skeletal muscle following a hyperinsulinemic-euglycemic clamp in male T1DM rodents using pre- and postclamp tissue analysis (40). This suggests that excess glucose is converted to lipid in skeletal muscle in T1DM through de novo lipogenesis (DNL) (91). Increased citrate synthase activity in T1DM rodents post-IVGTT may reflect DNL resulting from excess intracellular glucose. The increased glucose metabolism would significantly increase acetyl-CoA production, thereby overloading the Krebs cycle leading to excess citrate production by citrate synthase (92). Increased citrate can allosterically activate acetyl-CoA carboxylase to produce malonyl-CoA and promote lipid synthesis (92). Therefore, *β*-oxidation activity was assessed in the current study to investigate the ability of skeletal muscle to oxidize lipid formed through DNL during IVGTT, as this may impact insulin sensitivity (93). No differences in soleus muscle *β*-oxidation activity were observed between groups, suggesting that improvements in IR were unrelated to differences in lipid oxidation. Immediately following the IVGTT, it is plausible that glucose and lipid would “compete” for oxidation in skeletal muscle, such that lipid oxidation may be downregulated during periods of hyperglycemia (i.e., high glucose availability) (94, 95). Indeed, malonyl-CoA has been shown to limit fatty acid entry into the mitochondria for *β*-oxidation via inhibition of carnitine palmitoyltransferase-1 (92).

Since estradiol is associated with changes in insulin sensitivity and glucose and lipid metabolism (55, 96), we measured circulating serum levels of 17-*β* estradiol in all groups. We did not observe a significant difference in serum estradiol between groups and did not observe a strong explanation of variance in IVGTT AUC by estradiol, which suggests that circulating estradiol in these animals did not significantly influence glucose tolerance. Previous work has suggested that a subset of the female population with T1DM exhibits fluctuations in insulin sensitivity through the menstrual cycle, while other female patients do not (97–100). Our findings indicate a weak relationship between estradiol serum concentration and insulin sensitivity assessed by IVGTT in our female rodents with T1DM. Indeed, we have previously shown that estradiol concentration over the menstrual cycle likely does not play an important role in glycemic control during exercise in our female rodent model of T1DM (48).

### 4.1. Limitations

A limitation of the current study was the lack of pre-IVGTT tissue analysis to determine the effects of a glucose challenge on skeletal muscle metabolism, which limits the interpretation of the data. Specifically, it is unclear if white gastrocnemius muscle glycogen content was elevated at baseline or as a result of the IVGTT in DS animals. As such, mechanistic insights into the causative effects of intramyocellular glycogen content on insulin sensitivity in our female rodent model of T1DM remain unclear. Additionally, the use of a rat model to investigate the effects of T1DM on skeletal muscle metabolism may limit the transferability to patients due to the differences in the heterogeneity of muscle fiber composition between rodents and humans. For example, alterations in the metabolism of red and white muscle tissues reported in the current study may be more robust than in human patients. Furthermore, we utilized a preclinical rodent model of well-controlled T1DM, and therefore, our results provide insights exclusively into the effects of RT on skeletal muscle metabolism and IR in well-controlled female patients with T1DM using IIT. Finally, differences in the pre-IVGTT fasting period between T1DM and non-T1DM control animals (4 versus 12 hours, respectively) may have influenced intramuscular substrate content (e.g., glycogen) and confounded the results pertaining to insulin sensitivity and skeletal muscle metabolism.

## 5. Conclusions

The results of the current study demonstrate that female rodents with T1DM develop IR and that six weeks of RT prevent the decline in insulin sensitivity. In T1DM rodents, intramyocellular glycogen content in white gastrocnemius muscle is elevated but is prevented with RT independent of changes in circulating insulin levels, Akt signalling and muscle oxidative capacity associated with T1DM. This suggests that alterations in muscle glycogen storage may have a negative effect on insulin sensitivity in T1DM. While the role of RT in the mitigation of increased muscle glycogen storage is unclear, increased anabolic pathway flux to support muscle hypertrophy is a plausible mechanism which warrants future investigation. Taken together, these findings support the therapeutic role of RT as an effective intervention to improve IR in a female rodent model of T1DM.

## Data Availability

The datasets analyzed during the current study are available from the corresponding author on reasonable request.

## Supplementary Materials

Supplementary Materials Supplementary Figure 1: linear regression between insulin- (ng/ml) adjusted IVGTT AUC (mM∗minutes) and serum estradiol (ng/L) segregated by group. Abbreviations: AUC: area under the curve.

## References

[1] Singh V. P., Bali A., Singh N., Jaggi A. S. (2014). Advanced glycation end products and diabetic complications. *The Korean Journal of Physiology & Pharmacology: Official Journal of the Korean Physiological Society and the Korean Society of Pharmacology*.

[2] Subramanian S., Baidal D., Feingold K. R., Anawalt B., Boyce A. (2000). The management of type 1 diabetes. *Comprehensive Free Online Endocrinology Book*.

[3] Cleland S. J., Fisher B. M., Colhoun H. M., Sattar N., Petrie J. R. (2013). Insulin resistance in type 1 diabetes: what is ‘double diabetes’ and what are the risks?. *Diabetologia*.

[4] Donga E., van Dijk M., Hoogma R. P. L., Corssmit E. P., Romijn J. A. (2013). Insulin resistance in multiple tissues in patients with type 1 diabetes mellitus on long-term continuous subcutaneous insulin infusion therapy. *Diabetes/Metabolism Research and Reviews*.

[5] Šimonienė D., Platūkiene A., Prakapienė E., Radzevičienė L., Veličkiene D. (2020). Insulin resistance in type 1 diabetes mellitus and its association with patient’s micro- and macrovascular complications, sex hormones, and other clinical data. *Diabetes Therapy*.

[6] Koivisto V. A., Yki-järvinen H. (1990). Changes in muscle glucose metabolism in type 1 diabetes. *Annals of Medicine*.

[7] Pang T. T. L., Narendran P. (2008). Addressing insulin resistance in type 1 diabetes. *Diabetic Medicine*.

[8] Orchard T. J., Olson J. C., Erbey J. R. (2003). Insulin resistance–related factors, but not glycemia, predict coronary artery disease in type 1 diabetes. *Diabetes Care*.

[9] Specht B. J., Wadwa R. P., Snell-Bergeon J. K., Nadeau K. J., Bishop F. K., Maahs D. M. (2013). Estimated insulin sensitivity and cardiovascular disease risk factors in adolescents with and without type 1 diabetes. *The Journal of Pediatrics*.

[10] Schauer I. E., Snell-Bergeon J. K., Bergman B. C. (2011). Insulin resistance, defective insulin-mediated fatty acid suppression, and coronary artery calcification in subjects with and without type 1 diabetes: the CACTI study. *Diabetes*.

[11] Pozzilli P., Buzzetti R. (2007). A new expression of diabetes: double diabetes. *Trends in Endocrinology and Metabolism*.

[12] Kilpatrick E. S., Rigby A. S., Atkin S. L. (2007). Insulin resistance, the metabolic syndrome, and complication risk in type 1 diabetes. *Diabetes Care*.

[13] Cleland S. J. (2012). Cardiovascular risk in double diabetes mellitus--when two worlds collide. *Nature Reviews. Endocrinology*.

[14] Perseghin G., Lattuada G., Danna M. (2003). Insulin resistance, intramyocellular lipid content, and plasma adiponectin in patients with type 1 diabetes. *American Journal of Physiology-Endocrinology and Metabolism*.

[15] Cree-Green M., Stuppy J. J., Thurston J. (2018). Youth with type 1 diabetes have adipose, hepatic, and peripheral insulin resistance. *The Journal of Clinical Endocrinology and Metabolism*.

[16] Nadeau K. J., Regensteiner J. G., Bauer T. A. (2010). Insulin resistance in adolescents with type 1 diabetes and its relationship to cardiovascular function. *The Journal of Clinical Endocrinology and Metabolism*.

[17] Bergman B. C., Howard D., Schauer I. E. (2012). Features of hepatic and skeletal muscle insulin resistance unique to type 1 diabetes. *The Journal of Clinical Endocrinology*.

[18] Greenfield J. R., Samaras K., Chisholm D. J. (2002). Insulin resistance, intra-abdominal fat, cardiovascular risk factors, and androgens in healthy young women with type 1 diabetes mellitus. *The Journal of Clinical Endocrinology and Metabolism*.

[19] Merz K. E., Thurmond D. C. (2020). Role of skeletal muscle in insulin resistance and glucose uptake. *Comprehensive Physiology*.

[20] Kern M., Wells J. A., Stephens J. M. (1990). Insulin responsiveness in skeletal muscle is determined by glucose transporter (Glut4) protein level. *The Biochemical Journal*.

[21] Gregory J. M., Cherrington A. D., Moore D. J. (2020). The peripheral peril: injected insulin induces insulin insensitivity in type 1 diabetes. *Diabetes*.

[22] Monaco C. M., Perry C. G., Hawke T. J. (2017). Diabetic myopathy: current molecular understanding of this novel neuromuscular disorder. *Current Opinion in Neurology*.

[23] Gregory J. M., Kraft G., Scott M. F. (2015). Insulin delivery into the peripheral circulation: a key contributor to hypoglycemia in type 1 diabetes. *Diabetes*.

[24] Monaco C. M., Hughes M. C., Ramos S. V. (2018). Altered mitochondrial bioenergetics and ultrastructure in the skeletal muscle of young adults with type 1 diabetes. *Diabetologia*.

[25] Monaco C. M., Bellissimo C. A., Hughes M. C. (2020). Sexual dimorphism in human skeletal muscle mitochondrial bioenergetics in response to type 1 diabetes. *American Journal of Physiology-Endocrinology and Metabolism*.

[26] Dial A. G., Monaco C. M., Grafham G. K., Patel T. P., Tarnopolsky M. A., Hawke T. J. (2021). Impaired function and altered morphology in the skeletal muscles of adult men and women with type 1 diabetes. *The Journal of Clinical Endocrinology and Metabolism*.

[27] Monaco C. M., Tarnopolsky M. A., Dial A. G. (2021). Normal to enhanced intrinsic mitochondrial respiration in skeletal muscle of middle-to older-aged women and men with uncomplicated type 1 diabetes. *Diabetologia*.

[28] Minnock D., Annibalini G., Valli G. (2022). Altered muscle mitochondrial, inflammatory and trophic markers, and reduced exercise training adaptations in type 1 diabetes. *The Journal of Physiology*.

[29] Cree-Green M., Newcomer B. R., Brown M. S. (2015). Delayed skeletal muscle mitochondrial ADP recovery in youth with type 1 diabetes relates to muscle insulin resistance. *Diabetes*.

[30] Kacerovsky M., Brehm A., Chmelik M. (2011). Impaired insulin stimulation of muscular ATP production in patients with type 1 diabetes. *Journal of Internal Medicine*.

[31] Crowther G. J., Milstein J. M., Jubrias S. A., Kushmerick M. J., Gronka R. K., Conley K. E. (2003). Altered energetic properties in skeletal muscle of men with well-controlled insulin-dependent (type 1) diabetes. *American Journal of Physiology-Endocrinology and Metabolism*.

[32] Heyman E., Daussin F., Wieczorek V. (2020). Muscle oxygen supply and use in type 1 diabetes, from ambient air to the mitochondrial respiratory chain: is there a limiting step?. *Diabetes Care*.

[33] Kelley D., Mitrakou A., Marsh H. (1988). Skeletal muscle glycolysis, oxidation, and storage of an oral glucose load. *The Journal of Clinical Investigation*.

[34] Bruce C. R., Anderson M. J., Carey A. L. (2003). Muscle oxidative capacity is a better predictor of insulin sensitivity than lipid status. *The Journal of Clinical Endocrinology and Metabolism*.

[35] Dotzert M. S., McDonald M. W., Murray M. R., Nickels J. Z., Noble E. G., Melling C. J. (2018). Effect of combined exercise versus aerobic-only training on skeletal muscle lipid metabolism in a rodent model of type 1 diabetes. *Canadian Journal of Diabetes*.

[36] Yki-Järvinen H., DeFronzo R. A., Koivisto V. A. (1984). Normalization of insulin sensitivity in type I diabetic subjects by physical training during insulin pump therapy. *Diabetes Care*.

[37] Landt K. W., Campaigne B. N., James F. W., Sperling M. A. (1985). Effects of exercise training on insulin sensitivity in adolescents with type I diabetes. *Diabetes Care*.

[38] Hall K. E., McDonald M. W., Grisé K. N., Campos O. A., Noble E. G., Melling C. J. (2013). The role of resistance and aerobic exercise training on insulin sensitivity measures in STZ-induced type 1 diabetic rodents. *Metabolism*.

[39] Dotzert M. S., Murray M. R., McDonald M. W. (2016). Metabolomic response of skeletal muscle to aerobic exercise training in insulin resistant type 1 diabetic rats. *Scientific Reports*.

[40] Dotzert M. S., McDonald M. W., Olver T. D., Sammut M. J., Melling C. J. (2023). The influence of exercise training versus intensive insulin therapy on insulin resistance development in type 1 diabetes. *Journal of Diabetes and its Complications*.

[41] Reddy R., Wittenberg A., Castle J. R. (2019). Effect of aerobic and resistance exercise on glycemic control in adults with type 1 diabetes. *Canadian Journal of Diabetes*.

[42] Farinha J. B., Ramis T. R., Vieira A. F. (2018). Glycemic, inflammatory and oxidative stress responses to different high-intensity training protocols in type 1 diabetes: a randomized clinical trial. *Journal of Diabetes and its Complications*.

[43] Wallberg-Henriksson H., Gunnarsson R., Henriksson J. (1982). Increased peripheral insulin sensitivity and muscle mitochondrial enzymes but unchanged blood glucose control in type I diabetics after physical training. *Diabetes*.

[44] Brazeau A.-S., Rabasa-Lhoret R., Strychar I., Mircescu H. (2008). Barriers to physical activity among patients with type 1 diabetes. *Diabetes Care*.

[45] Yardley J. E., Sigal R. J. (2015). Exercise strategies for hypoglycemia prevention in individuals with type 1 diabetes. *Diabetes Spectrum: A Publication of the American Diabetes Association*.

[46] Alobaid A. M., Zulyniak M. A., Ajjan R. A., Brož J., Hopkins M., Campbell M. D. (2023). Barriers to exercise in adults with type 1 diabetes and insulin resistance. *Canadian Journal of Diabetes*.

[47] McDonald M. W., Murray M. R., Grise K. N. (2016). The glucoregulatory response to high-intensity aerobic exercise following training in rats with insulin-treated type 1 diabetes mellitus. *Applied Physiology, Nutrition, and Metabolism*.

[48] Larocque J. C., Gardy S., Sammut M., McBey D. P., Melling C. J. (2022). Sexual dimorphism in response to repetitive bouts of acute aerobic exercise in rodents with type 1 diabetes mellitus. *PLoS One*.

[49] Hasan S., Shaw S. M., Gelling L. H., Kerr C. J., Meads C. A. (2018). Exercise modes and their association with hypoglycemia episodes in adults with type 1 diabetes mellitus: a systematic review. *BMJ Open Diabetes Research & Care*.

[50] Yardley J. E., Sigal R. J., Perkins B. A., Riddell M. C., Kenny G. P. (2013). Resistance exercise in type 1 diabetes. *Canadian Journal of Diabetes*.

[51] Turner D., Luzio S., Gray B. J. (2015). Impact of single and multiple sets of resistance exercise in type 1 diabetes. *Scandinavian Journal of Medicine & Science in Sports*.

[52] McBey D. P., Dotzert M., Melling C. W. J. (2021). The effects of exercise training versus intensive insulin treatment on skeletal muscle fibre content in type 1 diabetes mellitus rodents. *Lipids in Health and Disease*.

[53] O’Neill C. C., Locke E. J., Sipf D. A. (2022). The effects of exercise training on glucose homeostasis and muscle metabolism in type 1 diabetic female mice. *Metabolites*.

[54] Jelenik T., Séquaris G., Kaul K. (2014). Tissue-specific differences in the development of insulin resistance in a mouse model for type 1 diabetes. *Diabetes*.

[55] Tramunt B., Smati S., Grandgeorge N. (2020). Sex differences in metabolic regulation and diabetes susceptibility. *Diabetologia*.

[56] Tarnopolsky M. A. (2008). Sex differences in exercise metabolism and the role of 17-beta estradiol. *Medicine and Science in Sports and Exercise*.

[57] Millstein R. J., Pyle L. L., Bergman B. C. (2018). Sex-specific differences in insulin resistance in type 1 diabetes: the CACTI cohort. *Journal of Diabetes and its Complications*.

[58] Derella C. C., Thomas J., Harris R. A. (2023). Women have greater endothelin-B receptor function and lower mitochondrial capacity compared to men with type 1 diabetes. *The Journal of Clinical Endocrinology and Metabolism*.

[59] American Diabetes Association (2018). 2. Classification and Diagnosis of Diabetes:Standards of Medical Care in Diabetes—2018. *Diabetes Care*.

[60] Carter S. L., Rennie C. D., Hamilton S. J., Tarnopolsky M. A. (2001). Changes in skeletal muscle in males and females following endurance training. *Canadian Journal of Physiology and Pharmacology*.

[61] Kielkopf C. L., Bauer W., Urbatsch I. L. (2020). Bradford assay for determining protein concentration. *Cold Spring Harbor Protocols*.

[62] Paquin J., Lagacé J.-C., Brochu M., Dionne I. J. (2021). Exercising for insulin sensitivity–is there a mechanistic relationship with quantitative changes in skeletal muscle mass?. *Frontiers in Physiology*.

[63] Poehlman E. T., Dvorak R. V., DeNino W. F., Brochu M., Ades P. A. (2000). Effects of resistance training and endurance training on insulin sensitivity in nonobese, young women: a controlled randomized trial. *The Journal of Clinical Endocrinology and Metabolism*.

[64] Holten M. K., Zacho M., Gaster M., Juel C., Wojtaszewski J. F., Dela F. (2004). Strength training increases insulin-mediated glucose uptake, GLUT4 content, and insulin signaling in skeletal muscle in patients with type 2 diabetes. *Diabetes*.

[65] Ahmadizad S., Haghighi A. H., Hamedinia M. R. (2007). Effects of resistance versus endurance training on serum adiponectin and insulin resistance index. *European Journal of Endocrinology*.

[66] Bucci M., Huovinen V., Guzzardi M. A. (2016). Resistance training improves skeletal muscle insulin sensitivity in elderly offspring of overweight and obese mothers. *Diabetologia*.

[67] Ibañez J., Izquierdo M., ARGuelles I. (2005). Twice-weekly progressive resistance training decreases abdominal fat and improves insulin sensitivity in older men with type 2 diabetes. *Diabetes Care*.

[68] Ismail A. D., Alkhayl F. F. A., Wilson J., Johnston L., Gill J. M., Gray S. R. (2019). The effect of short-duration resistance training on insulin sensitivity and muscle adaptations in overweight men. *Experimental Physiology*.

[69] Wilcox G. (2005). Insulin and insulin resistance. *Clinical Biochemist Reviews*.

[70] Gregory J. M., Smith T. J., Slaughter J. C. (2019). Iatrogenic hyperinsulinemia, not hyperglycemia, drives insulin resistance in type 1 diabetes as revealed by comparison with GCK-MODY (MODY2). *Diabetes*.

[71] Ueki K., Yamamoto-Honda R., Kaburagi Y. (1998). Potential role of protein kinase B in insulin-induced glucose transport, glycogen synthesis, and protein synthesis. *The Journal of Biological Chemistry*.

[72] Derave W., Hansen B. F., Lund S., Kristiansen S., Richter E. A. (2000). Muscle glycogen content affects insulin-stimulated glucose transport and protein kinase B activity. *American Journal of Physiology-Endocrinology and Metabolism*.

[73] Jensen J., Rustad P. I., Kolnes A. J., Lai Y.-C. (2011). The role of skeletal muscle glycogen breakdown for regulation of insulin sensitivity by exercise. *Frontiers in Physiology*.

[74] Danforth W. H., Harvey P. (1964). Glycogen synthetase and control of glycogen synthesis in muscle. *Biochemical and Biophysical Research Communications*.

[75] Danforth W. H. (1965). Glycogen synthetase activity in skeletal muscle. *The Journal of Biological Chemistry*.

[76] Jensen J., Jebens E., Brennesvik E. O. (2006). Muscle glycogen inharmoniously regulates glycogen synthase activity, glucose uptake, and proximal insulin signaling. *American Journal of Physiology-Endocrinology and Metabolism*.

[77] Munger R., Temler E., Jallut D., Haesler E., Felber J.-P. (1993). Correlations of glycogen synthase and phosphorylase activities with glycogen concentration in human muscle biopsies. Evidence for a double-feedback mechanism regulating glycogen synthesis and breakdown. *Metabolism*.

[78] Shannon C., Merovci A., Xiong J. (2018). Effect of chronic hyperglycemia on glucose metabolism in subjects with normal glucose tolerance. *Diabetes*.

[79] De Bock K., Derave W., Ramaekers M., Richter E. A., Hespel P. (2007). Fiber type-specific muscle glycogen sparing due to carbohydrate intake before and during exercise. *Journal of Applied Physiology*.

[80] Bally L., Buehler T., Dokumaci A. S., Boesch C., Stettler C. (2015). Hepatic and intramyocellular glycogen stores in adults with type 1 diabetes and healthy controls. *Diabetes Research and Clinical Practice*.

[81] Buehler T., Bally L., Dokumaci A. S., Stettler C., Boesch C. (2016). Methodological and physiological test–retest reliability of13C-MRS glycogen measurements in liver and in skeletal muscle of patients with type 1 diabetes and matched healthy controls. *NMR in Biomedicine*.

[82] He J., Kelley D. E. (2004). Muscle glycogen content in type 2 diabetes mellitus. *American Journal of Physiology-Endocrinology and Metabolism*.

[83] Staron R. S., Malicky E. S., Leonardi M. J., Falkel J. E., Hagerman F. C., Dudley G. A. (1990). Muscle hypertrophy and fast fiber type conversions in heavy resistance-trained women. *European Journal of Applied Physiology*.

[84] Staron R. S., Leonardi M. J., Karapondo D. L. (1991). Strength and skeletal muscle adaptations in heavy-resistance-trained women after detraining and retraining. *Journal of Applied Physiology*.

[85] Wackerhage H., Vechetti I. J., Baumert P. (2022). Does a hypertrophying muscle fibre reprogramme its metabolism similar to a cancer cell?. *Sports Medicine*.

[86] Larsen S., Scheede-Bergdahl C., Whitesell T., Boushel R., Bergdahl A. (2015). Increased intrinsic mitochondrial respiratory capacity in skeletal muscle from rats with streptozotocin-induced hyperglycemia. *Physiological Reports*.

[87] Del Prato S., Leonetti F., Simonson D. C., Sheehan P., Matsuda M., DeFronzo R. A. (1994). Effect of sustained physiologic hyperinsulinaemia and hyperglycaemia on insulin secretion and insulin sensitivity in man. *Diabetologia*.

[88] Item F., Heinzer-Schweizer S., Wyss M. (2011). Mitochondrial capacity is affected by glycemic status in young untrained women with type 1 diabetes but is not impaired relative to healthy untrained women. *American Journal of Physiology-Regulatory, Integrative and Comparative Physiology*.

[89] Parry H. A., Roberts M. D., Kavazis A. N. (2020). Human skeletal muscle mitochondrial adaptations following resistance exercise training. *International Journal of Sports Medicine*.

[90] Bergman B. C., Goodpaster B. H. (2020). Exercise and muscle lipid content, composition, and localization: influence on muscle insulin sensitivity. *Diabetes*.

[91] Aas V., Kase E. T., Solberg R., Jensen J., Rustan A. C. (2004). Chronic hyperglycaemia promotes lipogenesis and triacylglycerol accumulation in human skeletal muscle cells. *Diabetologia*.

[92] Dulloo A. G., Gubler M., Montani J. P., Seydoux J., Solinas G. (2004). Substrate cycling between _de novo_ lipogenesis and lipid oxidation: a thermogenic mechanism against skeletal muscle lipotoxicity and glucolipotoxicity. *International Journal of Obesity*.

[93] Zhang L., Keung W., Samokhvalov V., Wang W., Lopaschuk G. D. (2010). Role of fatty acid uptake and fatty acid *β*-oxidation in mediating insulin resistance in heart and skeletal muscle. *Biochimica et Biophysica Acta (BBA)-Molecular and Cell Biology of Lipids*.

[94] Hue L., Taegtmeyer H. (2009). The Randle cycle revisited: a new head for an old hat. *American Journal of Physiology-Endocrinology and Metabolism*.

[95] Jensen M. D. (2002). Fatty acid oxidation in human skeletal muscle. *The Journal of Clinical Investigation*.

[96] Hevener A. L., Zhou Z., Moore T. M., Drew B. G., Ribas V. (2018). The impact of ER*α* action on muscle metabolism and insulin sensitivity–strong enough for a man, made for a woman. *Molecular Metabolism*.

[97] Trout K. K., Rickels M. R., Schutta M. H. (2007). Menstrual cycle effects on insulin sensitivity in women with type 1 diabetes: a pilot study. *Diabetes Technology & Therapeutics*.

[98] Brown S. A., Jiang B., McElwee-Malloy M., Wakeman C., Breton M. D. (2015). Fluctuations of hyperglycemia and insulin sensitivity are linked to menstrual cycle phases in women with T1D. *Journal of Diabetes Science and Technology*.

[99] Gamarra E., Trimboli P. (2023). Menstrual cycle, glucose control and insulin sensitivity in type 1 diabetes: a systematic review. *Journal of Personalized Medicine*.

[100] Levy C. J., O’Malley G., Raghinaru D. (2022). Insulin delivery and glucose variability throughout the menstrual cycle on closed loop control for women with type 1 diabetes. *Diabetes Technology & Therapeutics*.

[101] Sammut M. J. (2023). 581-P: the effects of resistance training on insulin resistance development in female rodents with type 1 diabetes. *Diabetes*.

