# Supplementary material for: The Effects of Resistance Exercise Training on Skeletal Muscle Metabolism and Insulin Resistance Development in Female Rodents with Type 1 Diabetes
Source: J Diabetes Res. 2024 Feb 22;2024:5549762. doi: 10.1155/2024/5549762 (PMC10904684; doi:10.1155/2024/5549762)
Supplement: Supplementary Materials — Supplementary Figure 1: linear regression between insulin- (ng/ml) adjusted IVGTT AUC (mM∗minutes) and serum estradiol (ng/L) segregated by group. Abbreviations: AUC: area under the curve. [file 5549762.f1.docx]

**Supplementary figure 1.**

**Supplementary Figure 1.** Linear regression between insulin (ng/ml)-adjusted IVGTT AUC (mM*minutes) and serum estradiol (ng/L) segregated by group. Abbreviations: AUC, area under the curve.
